# Decrease in Haemoglobin in Association with Aneurysm Volume Loss after Endovascular Repair of Abdominal Aortic Aneurysm

**DOI:** 10.31083/j.rcm2407207

**Published:** 2023-07-17

**Authors:** Ying Li, Hanxi Zhang, Zhonghua Sun, Jun Zheng, Shangdong Xu, Lei Xu, Lizhong Sun, Yu Li

**Affiliations:** ^1^Department of Radiology, Beijing Anzhen Hospital, Capital Medical University, 100029 Beijing, China; ^2^Department of Radiology, The Seventh Affiliated Hospital of Sun Yat-sen University, 518000 Shenzhen, Guangdong, China; ^3^Discipline of Medical Radiation Science, Curtin Medical School, Curtin University, Perth, WA 6102, Australia; ^4^Department of Cardiac Surgery, Beijing Anzhen Hospital, Capital Medical University, 100029 Beijing, China; ^5^Department of Cardiac Surgery, DeltaHealth, 200336 Shanghai, China

**Keywords:** abdominal aortic aneurysm, anaemia, endovascular aneurysm repair, haemoglobin, computed tomography, analysis

## Abstract

**Background:**

Anaemia (low haemoglobin [Hb]) is well known to be associated with high mortality and adverse cardiac events following surgical treatment of abdominal aortic aneurysm (AAA). However, little is known about the relationship of AAA volume and Hb level alterations with endovascular repair of AAA. This study aimed to examine the changes in the Hb level and aneurysm volume before and after endovascular aneurysm repair (EVAR) for AAA and determine the relationship between the post-operative Hb level and the volume loss of AAA.

**Methods:**

This retrospective study reviewed patients with AAA who underwent EVAR between January 2020 and February 2021 at a tertiary medical centre. The clinical features and medical history of the patients were analysed. Alterations in the Hb level were recorded, and the AAA volume was calculated from computed tomography angiography images before and after EVAR to infer the association between the post-operative Hb level and alterations in AAA volume. Moreover, AAA volume, pre-operative Hb level and medical history were studied to identify the risk factors for anaemia 24 h after EVAR.

**Results:**

A total of 74 patients (mean age, 67 ± 7.9 years) were included in this study. The Hb level decreased significantly 24 h after EVAR, and the post-operative Hb level was negatively correlated with AAA volume loss (*r* = –0.252, *p* = 0.041). No relationship was observed between AAA volume loss and decrease in the Hb level (*r* = 0.072, *p* = 0.571) or between pre-operative AAA volume and decrease in the Hb level (*r* = 0.072, *p* = 0.566). Furthermore, 59.5% of the patients (n = 44) developed anaemia 24 h after EVAR. The odds ratio (OR) of anaemia 24 h after EVAR was 5.3 times higher in those with hypertension (OR, 5.304; 95% confidence interval [CI], 1.024–27.424) than in those without the condition. The pre-operative Hb level (or normal Hb level) was a protective factor (OR = 0.909; 95% CI, 0.853–0.969). The area under the receiver operating characteristic curve was 0.840. The post-operative Hb level declined significantly, and AAA volume loss contributed to it. Moreover, hypertension was identified to be a risk factor for anaemia 24 h after EVAR, and pre-operative Hb level was discerned to be a protective factor.

**Conclusions:**

The findings suggest that decrease in the Hb level in the early post-EVAR stage warrants the attention of surgeons, especially in patients with a history of hypertension or a low pre-operative Hb level.

## 1. Introduction

Abdominal aortic aneurysm (AAA) is common in the older population, and its prevalence ranges from 4% to 7% [1]. Endovascular aneurysm repair (EVAR) is a minimally invasive technique for treating AAA and exhibits the advantages of being less invasive and being associated with lower procedure-related mortality or complications compared with open surgery [2, 3, 4, 5].

Anaemia has been considered to be a part of the natural disease process in patients with AAA and was linked to peri-operative complications [6]. Previous studies have indicated that patients with pre-operative anaemia who undergo cardiac surgery have higher risks of developing adverse cardiac events than those without anaemia [7, 8]. Furthermore, the presence of a low haemoglobin (Hb) level or anaemia before EVAR has been reported to be associated with high mortality and adverse cardiac events [7, 9]. Diehm *et al*. [9] showed that long-term survival was significantly lower in patients with pre-operative anaemia than in those without anaemia and that the Hb level was inversely related to the maximum diameter of the AAA. Furthermore, AAA size has been documented to be independently associated with an increased risk of peri-operative complications [10]. During EVAR, the stent graft is firmly positioned at the upper and lower edges of AAA, which results in the narrowing of the lumen and indicates that the aneurysm is successfully excluded from systematic circulation. However, some blood is isolated outside the covered stent cavity, a process similar to acute haemorrhage, which causes a decrease in the Hb level or even anaemia [11]. With the advancements in segmentation software, the volumetric analysis of AAA has become feasible [12]. No study has so far reported post-operative Hb alteration or anaemia in relation to aneurysm volume change after EVAR.

Computed tomography (CT) is a routine imaging modality in the follow-up of patients with EVAR, and its primary role is to monitor the changes in the aneurysm size and detect endoleaks that occur commonly in patients after EVAR [13, 14, 15]. Usually, aneurysm diameter is measured and compared on pre- and post-EVAR CT images to determine the success of EVAR procedures. However, volumetric analysis has been shown to be more accurate than maximal diameter growth in determining whether surgical intervention is needed [16, 17]. In addition, a follow-up study has observed that lumen volume was superior to maximal diameter in predicting aneurysm enlargement [12].

This study aimed to investigate the alterations in the Hb level and the AAA volume (indicating the AAA lumen) before and after EVAR and examine the correlation between AAA volume loss and Hb level 24 h after the procedure. The attributes of AAA volume, pre-operative Hb level and history were analysed to determine the risk factors for anaemia 24 h after EVAR. Although several reports analysing various biomarkers in relation to their roles in EVAR outcomes have been published so far, this study adds valuable information to the existing literature by exploring the post-operative Hb level in relation to AAA volume change following EVAR. Most of the current studies have documented the association between pre-operative Hb level and EVAR outcomes and analysed AAA diameter changes.

## 2. Materials and Methods

### 2.1 Patients

A total of 296 patients from Beijing Anzhen Hospital, Beijing, China, were retrospectively examined between 31st January 2020 and 1st February 2021. AAA was defined as the presence of an abdominal aortic diameter of >3 cm or an enlargement 1.5 times the size of the normal abdominal aortic diameter [8]. Anaemia was defined as an Hb level of <130 g/L in men and <120 g/L in women in accordance with the guidelines of the World Health Organization [18]. Patients who had pre-operative anaemia, haemolytic disease, abnormal indirect bilirubin levels, complications of femoral artery puncture, renal impairment and incomplete images or missing information on the Hb level and did not undergo surgery or open repair surgery were excluded. Data on demographics, clinical features and medical history were obtained, and blood samples for analysing the Hb level were collected before EVAR and 24 h after the procedure. This study was approved by the Beijing Anzhen Hospital Ethics Committee (no. 2022023X).

### 2.2 Computed Tomography Angiography Images

Computed tomography angiography (CTA) was performed using four scanners, including two 128-slice CT (Somatom Definition Flash, Siemens Healthcare; Forchheim, Germany), one 256-slice CT (Revolution CT, GE Healthcare, Milwaukee, WI, USA) and one 320-slice CT (Aquilion One, Toshiba, Otawara, Japan), before EVAR and 7 days after it. The tube voltage was set at 100 kV or 120 kV, as automatically determined by the kV assist. The tube current was set at 320 mA with dynamic current modulation; collimation was set at 128- and 256-slice CT, 0.625 mm or 320-slice CT, 0.5 mm; and reconstruction slice thickness was set at 1 mm. All patients undergoing CTA were intravenously injected with a contrast agent (370 mg/mL of iodine, Ultravist, Bayer Schering Pharma, Berlin, Germany), and a scan was initiated when a 200 Hounsfield unit threshold was reached in the aorta. Bolus tracking was performed in the descending aorta at the level of the bifurcation of the trachea. The flow volume was 1.5 mL/kg, and the flow rate was 4–5 mL/s.

### 2.3 Imaging Analysis 

**Fig. 1.**
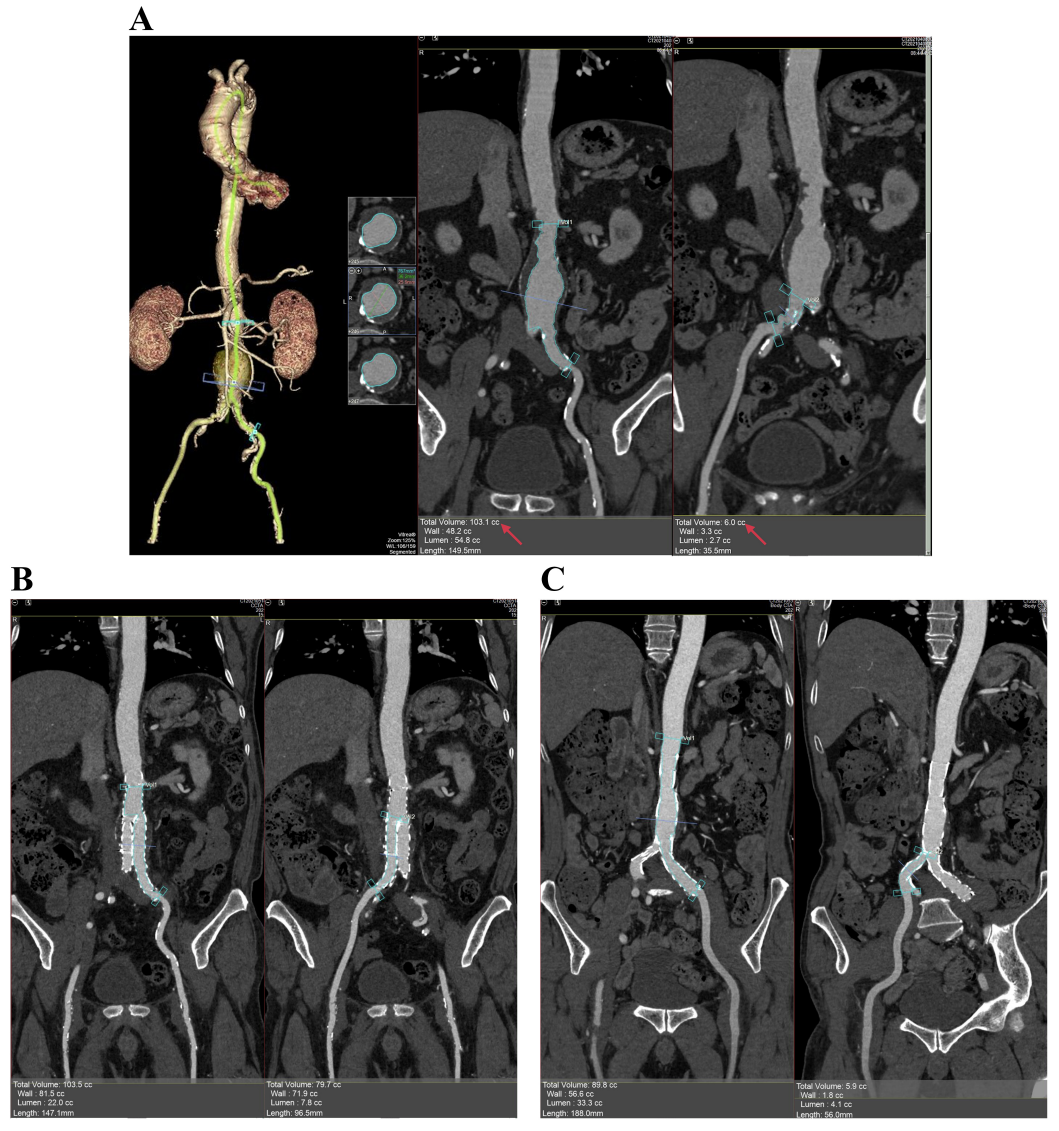
**Method of volume measurement**. (A) Volumes of the abdominal aortic aneurysm and bilateral iliac artery lumen before EVAR. (B,C) The volume of the intrastent (s) lumen at the level of aortic bifurcation (B) and at the level of common iliac arteries (C) after EVAR. EVAR, endovascular aneurysm repair.

All images were analysed using a separate workstation (Vitrea FX Workstation, Vital Images, Minnetonka, MN, USA). An experienced radiologist examined the aneurysm in three-dimensional multiplanar reconstruction (MPR) and orthogonal views and then edited the centerlines of the aorta and bilateral iliac arteries in the curved MPR view to verify the accuracy. The volume tool available for vessels was used (Vascular: Aorta Stent CT protocol). The click and drag option in the curved view was used to define the proximal and distal ends (from the lowest renal artery to the end of the left common iliac artery [volume 1] and from the bifurcation of the iliac artery to the end of the right common iliac artery [volume 2]). Subsequently, the volume of the lumen (VL) was determined (except for the thrombus regions, VL = V1 + V2). The same method was used to evaluate the intrastent volume (Vs) in the same segment after the surgery (Fig. 1). Here, AAA volume loss was defined as VL – Vs.

### 2.4 EVAR Procedure

During the EVAR, percutaneous puncture was used for aortic stent graft implantation in all patients. The puncture site was at the common femoral artery in all patients. Those who developed intraoperative complications, such as arterial rupture, ecchymosis, hematoma, pseudoaneurysm, arteriovenous fistula or post-operative endoleak following the EVAR procedure, were excluded.

### 2.5 Radiation Dose Measurements

CT dose length product (DLP) was recorded for each patient, and the effective dose was calculated by multiplying DLP with an organ coefficient factor of 0.014 mSv/mGycm for thoraco-abdominal CT angiography examination. The effective doses before and after the EVAR procedures were then compared to determine the presence of significant differences.

### 2.6 Statistical Analyses

Data were analysed using SPSS 26.0 (SPSS Inc., Chicago, IL, USA). Continuous variables were expressed as the mean (standard deviation, SD), and the pre- and post-EVAR Hb levels were compared using the paired *t*-test. A partial correlation analysis was performed to determine the relationship between post-operative Hb level and AAA volume loss, AAA volume loss and decrease in the Hb level and pre-operative AAA volume and Hb loss after adjusting for height, weight, hypertension and coronary heart disease. Binary logistic regression was performed to determine the predictors for anaemia 24 h after EVAR. Later, the receiver operating characteristic curve was plotted for assessing the prediction model. Differences were considered statistically significant at *p* < 0.05.

## 3. Results

### 3.1 Demographics, Clinical Features and Medical History

**Fig. 2.**
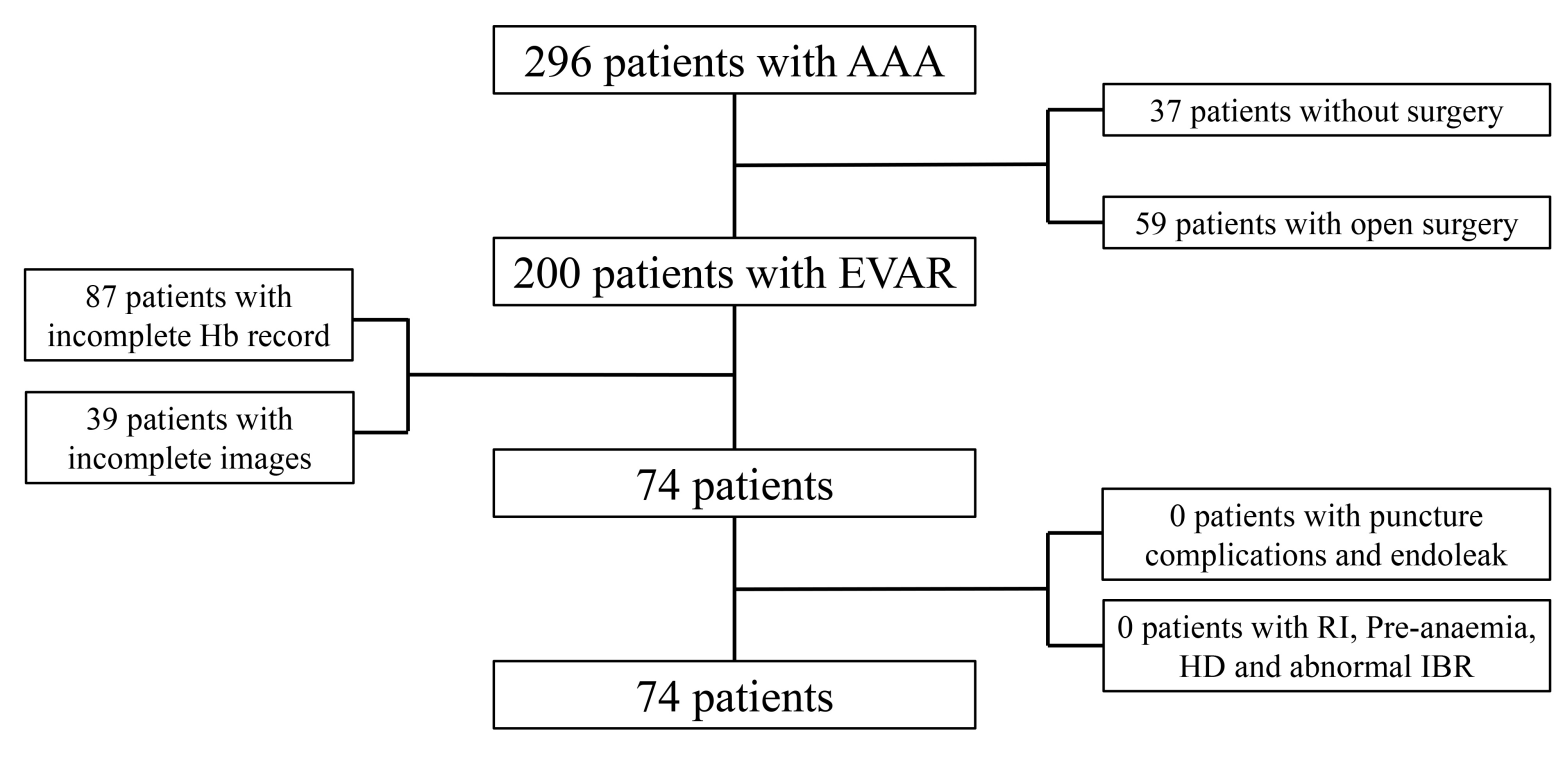
**Flowchart for the selection and analysis of patients with AAA (n = 74)**. After excluding patients lacking complete CTA or haemoglobin level data for pre- and post-EVAR, a total of 74 patients with AAA were included in the analysis. AAA, abdominal aortic aneurysm; CTA, computed tomography angiography; EVAR, endovascular aneurysm repair; RI, renal insufficiency; Pre-anaemia, pre-operative anaemia; HD, haemolytic disease; IBR, abnormal indirect bilirubin; Hb, haemoglobin.

Of the 296 patients with AAA, 37 who did not undergo surgery and 59 who underwent open repair surgery for other segments of the aorta or branch vessel lesion were excluded. Moreover, 87 patients with incomplete information on Hb levels and 39 patients with incomplete pre- or post-EVAR CTA images were excluded (Fig. 2). Finally, 74 patients (men, n = 69) with a mean age of 67 years were included in this study. The mean AAA diameter was 49 mm. Furthermore, 59.5% of the patients (n = 44) experienced anaemia 24 h after EVAR. Table 1 lists the patient characteristics.

**Table 1.** **Patient demographics of the study patients**.

Demographics		No. (%)
Sex (male)		69 (93.2%)
Age, mean (SD), y		66.6 (7.9)
Height, mean (SD), cm		170.3 (6.3)
Weight, mean (SD), kg		74.4 (10.5)
Medical history		
	Hypertension	54 (72.9)
	High cholesterol	48 (64.9)
	Diabetes mellitus	14 (18.9)
	Coronary heart disease	45 (60.8)
Mean AAA diameter (mm)		49.23 (13.41)
Post-operative anaemia		44 (59.5%)

AAA, abdominal aortic aneurysm; SD, standard deviation.

### 3.2 Hb Level, AAA Volume Alteration and Correlation Analysis 

**Fig. 3.**
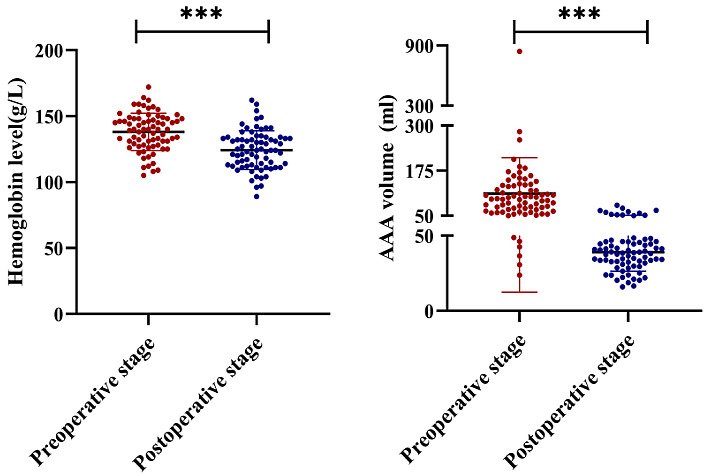
**Peri-operative and post-operative haemoglobin levels and AAA volumes**. The red colour indicates the pre-operative haemoglobin level and AAA volume, and the blue colour indicates the post-operative haemoglobin level and AAA volume. Note: ***, *p <* 0.001. AAA, abdominal aortic aneurysm.

**Fig. 4.**
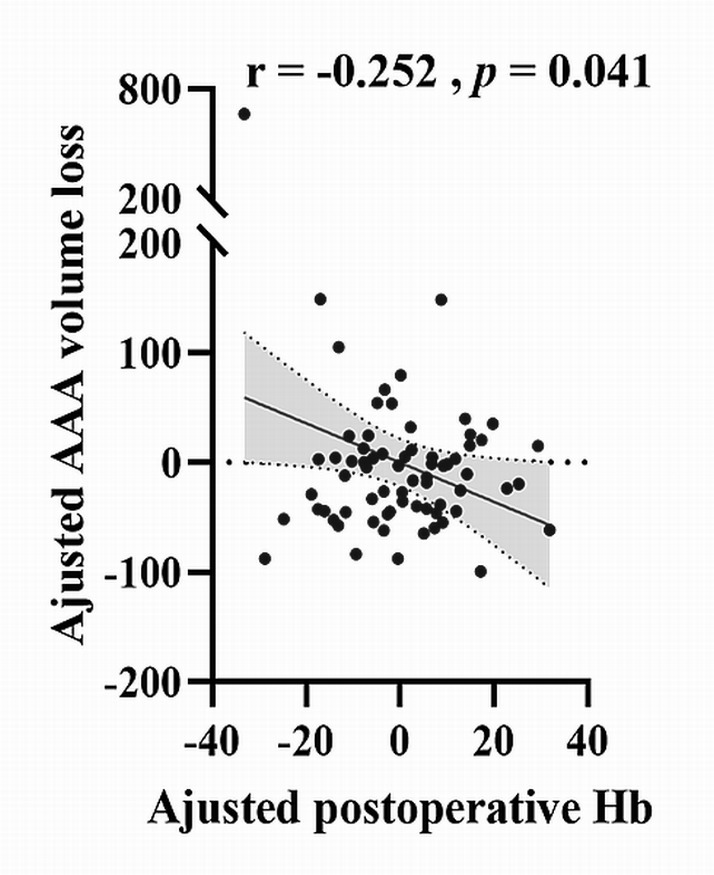
**Correlation between AAA volume loss and post-operative haemoglobin level**. The correlation analysis was performed between post-operative haemoglobin level and AAA volume loss (*r* = –0.252, *p* = 0.041) after adjusting for height, weight, hypertension and coronary heart disease. AAA, abdominal aortic aneurysm; Hb, haemoglobin.

**Fig. 5.**
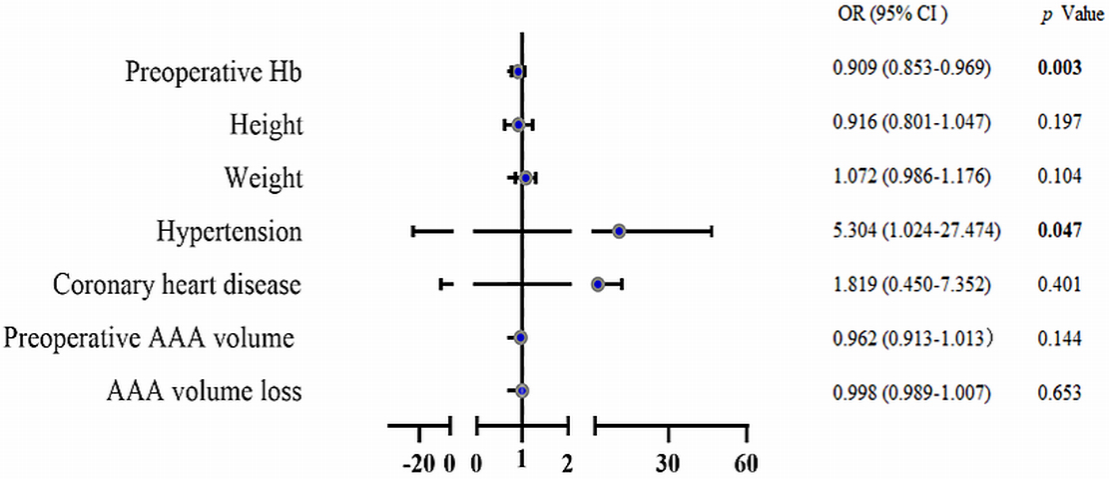
**Forest plot for post-operative anaemia**. The OR of anaemia 24 h after EVAR was 5.3 times higher for hypertension (OR, 5.304; 95% CI, 1.024–27.424) that for no hypertension, and the pre-operative haemoglobin level was a protective factor (OR = 0.909; 95% CI, 0.853–0.969). Odds ratio (OR) is shown with the 95% confidence interval (CI). EVAR, endovascular aneurysm repair; AAA, abdominal aortic aneurysm; Hb, haemoglobin. Bold data in the figure notes refer to statistically significant difference.

**Fig. 6.**
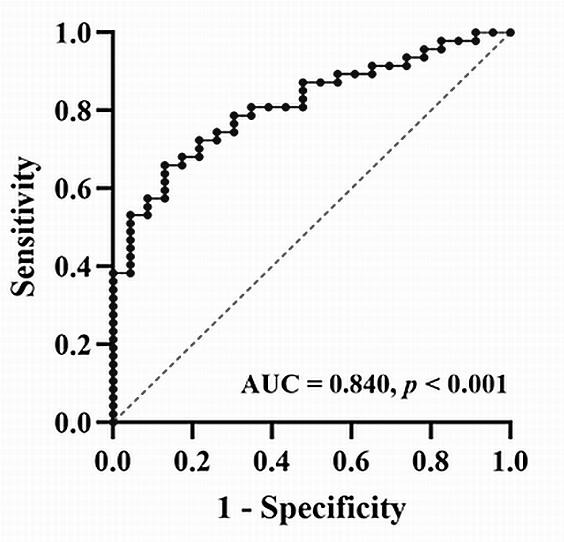
**Receiver operating characteristic curves of the binary regression model for predicting post-operative anaemia (AUC = 0.840, *p <* 0.001)**. AUC, area under the receiver operating characteristic curve.

The Hb level decreased significantly 24 h after EVAR (*p* < 0.001; Table 2 and Fig. 3). After adjusting for height, weight, hypertension and coronary heart disease, a negative correlation was noted between post-operative Hb level and AAA volume loss (*r* = –0.252, *p* = 0.041; Fig. 4). However, there was no correlation between AAA volume loss and decrease in the Hb level (*r* = 0.072, *p* = 0.571) or pre-operative AAA volume and Hb loss (*r* = 0.072, *p* = 0.566). Furthermore, for predicting anaemia 24 h after EVAR, the pre-operative Hb level was a protective factor (odds ratio [OR], 0.909; 95% confidence interval [CI], 0.853–0.969). The OR of anaemia 24 h after EVAR was 5.3 times higher in those with hypertension than in those without the condition (OR, 5.304; 95 % CI, 1.024–27.424; Table 3, Fig. 5). The area under the receiver operating characteristic curve for this model was 0.840, *p* < 0.001 (Fig. 6).

**Table 2.** **Pre- and post-operative AAA volume and haemoglobin level**.

	Pre-operative	Post-operative	*p* value
AAA volume, mean (SD), mL	111.6 (99.2)	39.2 (12.7)	<0.001
Haemoglobin level, mean (SD), g/L	138.1 (14.2)	124.3 (14.7)	<0.001

AAA, abdominal aortic aneurysm; SD, standard deviation.

**Table 3.** **Binary logistic regression for predicting post-operative anaemia**.

Factors	95% CI for OR		OR	*p*
	Lower	Upper		
Pre-operative Hb	0.853	0.969	0.909	**0.003**
Height	0.801	1.047	0.916	0.197
Weight	0.986	1.167	1.072	0.104
Hypertension	1.024	27.474	5.304	**0.047**
Coronary heart disease	0.45	7.352	1.819	0.401
Pre-operative AAA volume	0.913	1.013	0.962	0.144
AAA volume loss	0.989	1.007	0.998	0.653

OR, odds Ratio; CI, confidence interval; AAA, abdominal aortic aneurysm. Bold *p* values indicate statistically significant; Hb, haemoglobin.

### 3.3 Correlation Between Sex and Differences in Hb Levels

Further analysis was performed to compare the differences in Hb levels between men and women before and after the EVAR procedure. Table 4 shows the sex-based differences in pre-operative Hb levels, which were significantly higher in men than in women. However, no significant difference was noted in post-operative Hb levels.

**Table 4.** **Comparison of haemoglobin levels between men and women before and after EVAR**.

Hb values	Men (n = 69)	Women (n = 5)	*p* value
Pre-operative Hb	139 (13.9)	124.8 (13.7)	0.030
Post-operative Hb	124.8 (14.7)	117.4 (14.1)	0.280
Differences (mean/SD)	14.2 (12.2)	7.4 (11.7)	0.229

EVAR, endovascular aneurysm repair; Hb, haemoglobin; SD, standard deviation.

### 3.4 Comparison of Radiation Dose 

The mean effective doses for pre-operative and post-operative EVAR were calculated to be 5.05 ± 1.86 mSv and 6.61 ± 2.81 mSv, respectively, with the radiation dose increasing significantly after EVAR (*p* < 0.001).

## 4. Discussion

In this study, the Hb level declined significantly 24 h after EVAR, and 59.5% of the patients experienced anaemia. Moreover, the post-operative Hb level was negatively correlated with AAA volume loss. In the prediction of anaemia 24 h after EVAR, the results of binary logical regression showed that hypertension was a risk factor, whereas pre-operative Hb level was a protective factor. The area under the receiver operating characteristic curve for this model was 0.840.

In this study, the results demonstrated that the Hb level decreased or anaemia occurred after EVAR. This occurrence may be related to the process of EVAR as the aortic lumen is narrower than that before the procedure because of the formation of the new conduit. This conduit is introduced by the endovascular stent graft inside the aneurysm, where the aneurysm is excluded from systemic circulation and the blood between the aortic wall and the stent graft is isolated from circulation. This process is similar to acute haemorrhage. These findings are consistent with those from a previous study [19], which indicated that acute blood loss resulted in a decline in the Hb level or even anaemia. Moreover, according to the auto-resuscitation theory, after blood loss, the interstitial fluid moves into the capillaries to compensate for the decreased plasma volume [20]. This process begins at the onset of blood loss, and the plasma volume is replenished in a short time of 1 h [21, 22, 23]. Taken together, the Hb level decreases after blood loss, but the plasma volume is replenished by interstitial fluid compensation. This process results in a decrease in the Hb level.

Post-operative Hb decline or anaemia was observed after EVAR in this study, which agrees with a previous study [24]. Furthermore, the results showed that decrease in the Hb level after vascular surgery, including EVAR, is an independent risk factor for developing 30-day cardiovascular events. A decrease of 10 g/L in the Hb level was associated with a 20% increased risk of a 30-day cardiovascular event. The mean decrease in the Hb level in their study group was 22 g/L, whereas in our group, it was 14 g/L. Despite the relatively low decrease in the Hb level in our study and that by Valentijn *et al*. [24], when compared with the 50% decrease from baseline as shown by Karkouti *et al*. [25], the potential risk of post-operative decrease in the Hb level and its association with cardiovascular events should not be ignored. Blood transfusions are beneficial in preventing mortality and morbidity in patients who suffer from intraoperative haemorrhage and in those who are severely anaemic in the peri-operative period [26]. The recommended threshold for red blood cell transfusion is Hb level of <70 g/L [27]. A previous study found that patients with mild anaemia receiving peri-operative transfusion had significantly higher odds of mortality and in-hospital complications (ORs of 5.7 and 4.3, respectively) than those who did not receive transfusion (mild anaemia was defined as a Hb level of 100–120 g/L in women and 100–130 g/L in men) [28]. In addition, another study reported that transfusions were rarely beneficial when Hb levels were >100 g/L [29]. In our study, the mean post-operative Hb level was 124 g/L, which exceeded 100 g/L and did not meet the blood transfusion criteria. Thus, we inferred that transfusions would not benefit the patients. In sum, the significance of anaemia or decreased Hb level should not be neglected because it is a reliable marker for predicting the risk of post-operative cardiac events, yet transfusion could make it worse when the level is >100 g/L.

Furthermore, our results indicated that post-operative Hb level was negatively correlated with AAA volume loss. In addition, no correlation was observed between AAA volume loss and decrease in the Hb level or pre-operative AAA volume and decrease in the Hb level. This lack of correlation could be ascribed to the following reasons: First, as per the auto-resuscitation theory mentioned above, after blood loss, the interstitial fluid moves into the capillaries to compensate for the decrease in the plasma volume [20]. This compensation ability varies from patient to patient. In addition, the fluid infusion and urine volume during the surgery differ among patients. This may be the reason for the absence of correlation between AAA volume loss and decrease in the Hb level. Second, the AAA volume loss depends on the volume of the aneurysm and the size of the stent, which is determined by its diameter and length. In general, the diameter of the stent is based on the diameter of the aneurysm neck. Therefore, a greater pre-operative AAA volume would be associated with more blood loss only if the diameter of the aneurysm neck and the length of the stent are similar. These findings suggest that aneurysm volume loss may be responsible for a lower post-operative Hb level 24 h after EVAR.

Finally, the predictor model results showed that hypertension was a risk factor for anaemia 24 h after EVAR. A previous study indicated that hypertension is a risk factor for cardiovascular disease [30] and AAA because high blood pressure causes an increase in the diameter [31]. Most AAAs contain a complex structure of fibrin, inflammatory cells, platelets and red blood cells in the aneurysmal sac, known as intraluminal thrombus [32]. An association is present between endothelial dysfunction and hypertension [33]. Endothelial dysfunction may lead to tissue swelling, chronic inflammation and thrombosis [34]. During thrombosis, red blood cells in the circulating blood decrease, leading to a decrease in the Hb level. In addition, according to Bernoulli’s principle, as the pressure inside the aneurysm increases, the velocity of the flow decreases [35]. This slow blood flow would result in more blood isolating outside the graft during EVAR. Furthermore, from the pathological perspective, during hypertension, blood vessels become stiffer owing to distension [36]. This decreased vascular elasticity causes more blood to remain in the vessel, especially in the aneurysm, which can augment the pressure and further reduce the blood flow. Thus, hypertension would result in more blood being isolated outside the graft, thus leading to increased blood loss during EVAR.

The effect of radiation dose on red blood cell counts and Hb level alterations remains to be determined. Previous studies reported that red blood cell counts and Hb levels decreased significantly as a result of prolonged radiation exposure [37, 38]. However, Tian *et al*. [39], in their recent study, analysed red blood cell counts and Hb level alterations in medical workers with exposure to low-dose radiation and found no significant changes in red blood cell counts and Hb levels in both sexes with extended service. Our results showed significant increase in the radiation dose following EVAR treatment, but the Hb levels decreased 24 h after the procedure. Thus, increased radiation dose may not contribute to the decrease in Hb levels owing to the fact that the Hb recording time was earlier than that of CT scanning.

The strengths of this study are as follows: (1) The idea was innovative as we correlated the post-operative Hb level with the AAA volume loss after EVAR. Furthermore, we identified the risk factor for post-operative anaemia 24 h after EVAR. (2) The cost of the research is low because of its retrospective nature.

However, this study has some limitations that should be addressed. First, the number of patients was small. In the future study, we intend to enrol more patients. Moreover, the mean AAA diameter was <50 mm in this study group. Inclusion of AAA cases with a larger diameter is necessary to validate our findings. Second, the urine volume and infusion administration were not calculated in detail because of the retrospective nature of this study. The operation record was not complete, but complete surgical records containing data on these two volumes were roughly equal during the procedure. Third, we did not follow-up patients in this group regardless of the presence of anaemia 24 h after EVAR. This gap needs to be addressed in future studies preferably by comparing major adverse cardiac events in patients who developed anaemia with those who did not. A previous study signified that the decrease in Hb levels in the first 24 h after the surgery (Day 0 to Day 1) underestimated the ultimate lowest Hb level on Day 2. A further decrease of 4.4 g/L in Hb levels was noted between Day 1 and Day 2, but no significant changes were found from Day 2 to Day 3, Day 3 to Day 4 and Day 4 to Day 5 [40]. Therefore, future work should investigate whether the degree of Hb decline is more severe in patients with anaemia in the first 24 h after EVAR and whether they are likely to develop chronic anaemia following EVAR treatment.

## 5. Conclusions

Our results indicate that the Hb level decreased and even reached the diagnostic level for anaemia 24 h after EVAR. Furthermore, the post-operative Hb level was negatively correlated with the aneurysm volume loss. For predicting anaemia 24 h after EVAR, a regression model was developed, which indicated that history of hypertension was a risk factor and that the pre-operative Hb level exerted a protective effect. The model suggested that aneurysm volume loss is worthy of attention owing to its negative association with the Hb level 24 h after EVAR. Furthermore, history of hypertension should be included in the post-operative risk assessment of patients with AAA undergoing EVAR treatment as it is a risk factor for anaemia 24 h after the procedure.

## Data Availability

The datasets generated and/or analyzed during the current study are not publicly available due to ethical restrictions but are available from the corresponding author on reasonable request.

## Abbreviations

AAA, abdominal aortic aneurysm; AUC, area under the curve; CI, confidence interval; CTA, computed tomography angiography; DLP, dose length product; EVAR, endovascular aneurysm repair; Hb, haemoglobin; OR, odds ratio; VL, volume of the lumen.
